# A Comparative Perspective on the Cerebello-Cerebral System and Its Link to Cognition

**DOI:** 10.1007/s12311-022-01495-0

**Published:** 2022-11-23

**Authors:** Neville Magielse, Katja Heuer, Roberto Toro, Dennis J. L. G. Schutter, Sofie L. Valk

**Affiliations:** 1 Institute of Neuroscience and Medicine (INM-7: Brain and Behaviour), Research Center Jülich, Jülich, Germany; 2 Otto Hahn Cognitive Neurogenetics Group, Max Planck Institute for Human Cognitive and Brain Sciences, Leipzig, Germany; 3 Institute of Systems Neuroscience, Heinrich Heine University, Düsseldorf, Germany; 4 Institute Pasteur, Unité de Neuroanatomie Appliquée et Théorique, Université Paris Cité, Paris, France; 5 Department of Neuropsychology, Max Planck Institute for Human Cognitive and Brain Sciences, Leipzig, Germany; 6 Experimental Psychology, Helmholtz Institute, Utrecht University, Utrecht, The Netherlands

**Keywords:** Cerebellum, Cerebral cortex, Cognition, Evolution, Human, Primates

## Abstract

The longstanding idea that the cerebral cortex is the main neural correlate of human cognition can be elaborated by comparative analyses along the vertebrate phylogenetic tree that support the view that the cerebello-cerebral system is suited to support non-motor functions more generally. In humans, diverse accounts have illustrated cerebellar involvement in cognitive functions. Although the neocortex, and its transmodal association cortices such as the prefrontal cortex, have become disproportionately large over primate evolution specifically, human neocortical volume does not appear to be exceptional relative to the variability within primates. Rather, several lines of evidence indicate that the exceptional volumetric increase of the lateral cerebellum in conjunction with its connectivity with the cerebral cortical system may be linked to non-motor functions and mental operation in primates. This idea is supported by diverging cerebello-cerebral adaptations that potentially coevolve with cognitive abilities across other vertebrates such as dolphins, parrots, and elephants. Modular adaptations upon the vertebrate cerebello-cerebral system may thus help better understand the neuroevolutionary trajectory of the primate brain and its relation to cognition in humans. Lateral cerebellar lobules crura I-II and their reciprocal connections to the cerebral cortical association areas appear to have substantially expanded in great apes, and humans. This, along with the notable increase in the ventral portions of the dentate nucleus and a shift to increased relative prefrontal-cerebellar connectivity, suggests that modular cerebellar adaptations support cognitive functions in humans. In sum, we show how comparative neuroscience provides new avenues to broaden our understanding of cerebellar and cerebello-cerebral functions in the context of cognition.

## Introduction

Contemporary views on human brain evolution and the neural correlates of internally oriented mental functions such as cognitive control, autobiographical memory, and social cognition put special emphasis on the cerebral cortex [1] and its massive expansion over the primate lineage [2, 3]. In support of this notion, empirical studies have established the involvement of the human cerebral cortex, and in particular its association cortices, in a wide array of cognitive functions [4, 5], and revealed complex changes to this system during primate evolution [6]. As a result of the cognitive neurosciences focusing on the cerebral cortical basis of mental functions, researchers may have overlooked the importance of another brain structure: the cerebellum.

The adult human cerebellum accounts for approximately 10% of the total brain volume [7–9]. Despite its modest size, the cerebellum contains approximately 80% of all neurons in the human brain [9]. Cerebellar granule cells (CGCs), the most numerous nerve cells in the brain, make up most of these neurons. CGCs have four short dendrites on average and are known to receive several different synaptic inputs [10, 11].

The cerebellum has traditionally been viewed as a brain region dedicated to motor-related functions [12]. However, accumulating evidence shows cerebellar involvement in cognitive, affective, and social processes including (verbal) working memory, decision making, Theory of Mind, social mirroring and mentalizing, and emotional processing [13–15]. The cerebellum is proposed to form internal models across intrinsic functional connectivity networks [16, 17]. The idea that the cerebellum contributes to non-motor functions is not new. Early cerebellar lesion studies showed cognitive alterations in patients [18, 19], followed by theories of — potentially analogous — cerebellar contribution across functional domains [20–23], theories that built upon the Marr-Ito-Albus [24–26] model of cerebellar motor learning and control [27]. The foundation for theories of a cognitive role for the cerebellum and the Marr-Ito-Albus model both lie within the particularly organized structure of the cerebellar cortex [28, 29]. As pointedly summarized by Lazaros Triarhou [30], Sven Ingvar described already in 1918 how the organization of the cerebellum is almost identical across vertebrates into the minutest details [31]. Ariëns Kappers, along with Carl Huber and Elizabeth Crosby, provided early explanations of the course of phylogenetic development of the vertebrate nervous system. The work includes many theoretical accounts, structure–function correlations, and biological explanations for observed cross-species anatomical differences [32]. Olof Larsell further established cerebellar lobular nomenclature and revealed cross-species homology in lobules, folia, and fissures by describing developmental forms. This work is best summarized in the books of Olof Larsell and Jan Jansen [33, 34]. The comparative work of Rudolf Nieuwenhuys should also be mentioned here, as it for example showed that cerebellar nuclei — an important relay of the cerebello-cerebral system (CCS) — stem from the cerebellar anlage across all vertebrates [35]. The extensive studies by Jan Voogd and Mitchell Glickstein further illustrate morphological similarities across vertebrates, but also underscore differences in external shape, neuron distribution, and prominence of distinct longitudinal zones and connectivity [36, 37], relating these similarities and differences to cerebellar functions [38].

The aim of the present review is to provide an updated [39] comparative evolutionary account that supports cerebellar involvement in primate cognitive functions. We will discuss literature supporting the theory that the interconnected cerebellum and cerebral cortex have evolved in tandem to support mental functions, focusing on primates. Moreover, we consider how in several vertebrates beyond primates, cerebello-cerebral structures have coevolved with cognitive ability. This shows that the expansion of the CCS may provide a parsimonious neurobiological substrate supporting both motor and non-motor functions [40]. Additionally, we describe how contemporary neurosciences support the role of the cerebellum in human cognition, specifically. We describe open questions on cerebellar function, and how contemporary neuroscientific tools may be used alongside the comparative method to answer these questions. Altogether, we aim to deepen appreciation of cerebellar functional diversity and how it is studied, which might ultimately prove important in understanding human brain function in the context of health and disease.

## Origin of Size-Based Inferences About Human Brain Uniqueness

Brain size has long been an important tool to study what makes the human brain able to contribute to diverse functions such as cognition, language, and motor functions. The human encephalization quotient (EQ), representing relative brain to body size, is seven versus other mammals, and three versus other primates [41–43]. Within the genus *Homo*, Neanderthals and more modern species have some of the highest EQs, but have reached these through distinct evolutionary trajectories [44]. High encephalization in contemporary humans has been interpreted as an important feature enabling increased ability to perform internally oriented mental processes. This notion is rooted in the idea that a higher brain-to-body ratio decreases the proportion of the brain dedicated to control of the body, freeing up brain space for more cognitively demanding, flexible, and multimodal sensory processes [45]. Indeed, in elephants and whales, that have much larger brains than humans, more brain tissue is dedicated to bodily functions [43, 46]. However, the proposed relation between high EQ and cognitive ability fails to explain the relationship between, for example, capuchins and gorillas, whose EQs and cognitive ability show a double dissociation [43, 47]. Additionally, in larger brains the effect of having a higher EQ should necessarily be more pronounced [48]. Subsequently, it was claimed that the best predictor of cognitive ability is brain size itself [47]. However, the implicit assumption on which this relationship rests, namely that neuron number and brain size scale in the same manner across species, has been falsified [49–52]. Therefore, if we consider that neuron number — and not total brain volume including glia and neuropil — is the most important parameter, it becomes clear that considering brain size may tell us just part of the story [48]. While absolute and relative brain size increase in humans, neuronal and non-neuronal cell numbers do not differ from the general primate trend [8, 9]. When studying brain size, it is also important to consider intraspecific variability and its relation to cognition. Within humans, general intelligence [53] may [54] or may not [55] correlate with brain size. Although intraspecific variability in cognition may be caused by mechanisms distinct from those causing evolutionary variation [56], it is important to consider brain size variations within species. For example, in the great tit (*Parus major*), intraspecific variation in problem-solving ability correlates with reproductive success [57], showing that variability in cognitive abilities may shape evolutionary trajectories.

Brain reorganization may be more relevant to anthropoid evolution than increases in brain size [58], and different volume-to-neuron number scaling rules may apply across species [49, 50, 52]. While brain size increases exponentially with the number of neurons in rodents [49], this relation is almost linear in primates [52]. Assuming that the total number of neurons is related to cognitive ability, this scaling principle may suggest that primates have a more economical brain organization [48, 59, 60]. Different components of the brain may furthermore scale at different rates [61]. For example, the relative number of neurons in the cerebrum and cerebellum differs vastly between humans and elephants [51]. For that reason, it is important to consider the scaling of the human brain in the context of non-human primates (NHPs), as to reveal what relative and specific changes have occurred. Due to extensive shared evolutionary history, primate brains are more likely to fall within the same taxon cerebrotype [62, 63], referring to the unique and coordinated patterns of brain evolution that have developed for groups of species [64], among which reorganization [58] and mosaic adaptations [61] can be examined. Performing comparative analysis within rather than between taxon-cerebrotypes prevents unjust generalization when comparing one cerebrotype to another [56, 64].

At the same time, care should be taken in respect to the assumption that associated cognitive abilities in the primate clade have a single evolutionary origin. Firstly, other species such as whales and corvids, although they may show divergent neuroanatomical evolution, may nonetheless show similar cognitive abilities, such as causal reasoning and mental time travel [65]. Describing similarities in CCS composition across vertebrates may provide evidence for the role of the system in cognition generally. Secondly, even between chimpanzees and humans there are independent evolutionary trajectories: for example, gyri including the inferior pre- and postcentral gyrus and distinct neurocranial features such as the cerebellar fossa have shifted their locations [66]. Endocast data from extinct hominid species can be used to strengthen inferences about evolutionary changes made from extant NHP-species, provided these inferences are supported by sulcal imprints [66].

### Evolutionary Theories Are Not Mutually Exclusive

Before considering the evolution of the CCS, we highlight several theories in comparative neuroscience for describing allometry that will help put many of the findings reported in this review in perspective. Initially, two main theories were formulated: developmental constraint [67] and mosaic evolution [61]. In the developmental constraint model, regional changes are thought to be strongly coordinated due to conserved neurodevelopmental mechanisms across the brain that cause coordinated scaling. Strong allometric patterns are often taken as evidence for developmental constraints [67, 68]. Furthermore, the model posits that the basic brain scaling pattern may have already evolved early in vertebrate brain evolution and that brain mass may have increased and decreased without compromising function [68]. The mosaic evolution theory instead suggests that specific selection may take place in areas that confer behavioral capacities, allowing functional modules to expand relatively independently from the rest of the brain in a mosaic fashion [61]. The mosaic theory has developed into the functional constraint theory [69], positing that functional systems must maintain allometric relationships to perform their functions. Since, an alternative adaptationist approach has been suggested, which posits that species adaptations are not only responsible for deviations from the allometric trend but are the main mechanism behind it [70, 71]. It is likely that neither the developmental nor functional constraint theory will be able to fully explain primate brain evolution [71], and it is yet unclear whether the predictions made by the adaptationist approach will hold true in primate comparative data. However, there is value in considering whether these theories may be supported by comparative data [69]. Therefore, we will use these complementary theories to help guide our understanding of primate CCS evolution.

### Neocortical Scaling Underlines the Need for Connectivity-Related Comparison

Contrary to previous reports [72, 73], the human neocortex may not have evolved exceptionally over the primate lineage in terms of its volume [74, 75]. Much of the organization of the human neocortex is predictable based on the general primate trend, such as the neuron number [8, 9], and relative neocortical white-to-gray matter volumes [72, 76]. As measured by neuron and glia cell number allometric trends in primates, the human brain may just be an allometrically scaled-up primate brain [48]. At the level of the entire neocortex, both volumetric measures and cell counts do not provide evidence for human specialization. One may need to focus on more specific adaptations such as cell type innovations [77], altered organization [78–80], or comparative analysis in relation to functional topography and connectivity to detect primate or human adaptations [61].

In particular, the CCS [81, 82] may have undergone specific modular adaptations supporting non-motor operation in primates [61, 73], as well as in cetaceans [83] and parrots [84]. Now, with elaborate phylogenetic techniques and additional neuroscientific tools, investigation of the non-motor CCS can be further substantiated. We consider here how the CCS may have generally been involved in supporting non-motor demands during vertebrate evolution, paving the way for specific modular adaptations over the primate lineage.

## Cerebello-Cerebral Connectivity as an Anatomical Blueprint for Evolutionary Adaptation

The CCS has been proposed to be among the evolutionary correlates of non-motor functions across a wide range of species [20, 83–86]. In primates, the system has undergone distinct adaptations. There are various arguments underlining the relevance of this system for human cognition [13]. The CCS shows a characteristic evolutionary trajectory in anthropoids [58]. In humans, the cerebellum is structurally [87, 88] and functionally [89, 90] connected to cerebral association cortices, including PFC and temporoparietal cortices, that are involved in cognitive networks [91]. Crucially, the components of the primate CCS show correlated evolutionary expansion that can be predicted by their connectivity [85, 86]. In the primate CCS, neuron numbers also coevolve. Specifically, the numbers of neurons in the cerebellum and cerebral cortex scale predictably with approximately four new cerebellar neurons for every cerebral neuron [7]. This shows that brain size and neuron number scale together in the system’s components, which is important when using CCS structure size as a proxy for cognitive ability. Furthermore, this observation argues for the evolution of the CCS being constrained by connectivity. A recent study revealed that the size of the CCS coincides with the increase of brain size in primates [40]. This suggests that the association between primate brain size increase and cognitive ability [45, 47] may be linked to the volumetric increase of the CCS.

The CCS did not develop as a non-motor module in primates exclusively, instead divergently evolving in diverse vertebrates, including those with high performance on tasks measuring abstract reasoning abilities [92–95]. The cerebellum and cerebrum scale highly predictably and are enlarged in cetaceans [83]. As the cetacean CCS makes up most of the brain, and their encephalization can be predicted by social structure and group size (albeit non-linearly), it is not unreasonable to suspect involvement of the CCS in socio-cognitive functions. Differences in relative cerebellar-to-whole brain volumes in killer whales (14%) and sperm whales (7%) [96], and relative lateral — versus medial — cerebellar expansion [97] as well as higher cerebellum-to-body ratios in dolphins compared to other cetaceans [98] illustrate modular organization within the CCS. Differences in relative cerebellar volumes may stem from ecological factors [96, 99] that may be related to cognitive ability [96, 98]. Parrots have also developed a primate-like CCS [84]. This raises the question whether the CCS is also involved in supporting abstract cognitive abilities in parrots, such as logical reasoning [93], as the CCS is suited for supporting non-motor functions [13, 20, 21, 100, 101]. These functions may further include grasping of abstract concepts, mimicry, language or language-like communication, and awareness of the self [92], which are present in a wide range of cetaceans, birds, and primates. Moreover, corvids are significantly more likely to display play behavior than other birds [102]. In primates, play time budget correlates with the size of the CCS, and most strongly with cerebral non-prefrontal transmodal association grey matter and the posterior cerebellum [103]. This substantiates the proposed role of play in shaping diverse socio-cognitive abilities through specific adaptations of the CCS [103]. Future research may look to establish clear associations between the CCS and cognitive capacities.

Lastly, Shine and Shine provide a theoretical account, namely that the interconnected CCS, including the basal ganglia [104–106], are a suitable neuroanatomical substrate for automatization of behaviors [100]. The authors further describe how bipedality has potentially accelerated a shift of relative brain activity from the cerebral cortex to the cerebellum during learning in humans, supporting this automatization [100]. Due to extensive functional modular organization of the CCS including the basal ganglia [81, 101, 107–109], such a shift may extend to the cognitive domain across vertebrates [20, 21, 100]. Automatization frees up cognitive resources, which may be beneficial for complex socio-cognitive behaviors [110].

Altogether, the vertebrate CCS may offer an ideal substrate for supporting cognition across species. Next, we will describe the general anatomy of the primate CCS, after which we list evidence of adaptations in the cerebellum and cerebral association areas over the primate lineage. These adaptations may specifically benefit from preexistent CCS anatomy [81] and physiology [108] to contribute to emergent cognitive abilities.

### Anatomy of the Primate Cerebello-Cerebral System

The primate CCS, as revealed in NHPs through invasive tracing methods [111], consists of distinct reciprocal closed loops that connect the cerebellum to the cerebral cortex [81]. The cerebellum-to-cerebral cortex efferents primarily pass through the cerebellar nuclei, the superior cerebellar peduncle (*brachium conjunctivum*), and the thalamus to reach distinct cerebral cortical areas. The cerebral cortex projects back to the cerebellum through anterior pontine nuclei (APN) [81, 111]. In *Cebus apella* (tufted capuchin, a New World monkey), reciprocal closed loops separate motor and non-motor faculties into dorsal and ventral parts of the dentate nucleus (DN) and thalamus [112, 113], mirroring distinct functional topography in the cerebellar and cerebral cortex [39, 81, 114]. Leiner, Leiner, and Dow first described how such modular organization of the CCS may have allowed the cerebellum to contribute to, for example, cognitive and language functions [82]. Anatomical studies revealed that the primate neocerebellum communicates reciprocally with parietal, temporal, and prefrontal association areas, as well as Broca’s area, through the DN and ventrolateral thalamus (VL), and pontine nuclei, respectively [82]. In more recent years, the advent of advanced tractography methods including diffusion-weighted imaging has allowed the modeling of crossing fiber bundles across the entire brain in vivo [87, 88], revealing the anatomical organization of the CCS in live humans. The cerebello-thalamo-cerebral (CTC) pathway, the cerebellar efferent pathway, was shown to involve the cerebellar cortex, DN, superior cerebellar peduncle, red nucleus, the ventrolateral thalamus, and lastly contralateral cerebral cortex. Furthermore, most prominent streamlines were found in the posterolateral crura I-II and lateral lobules VIIb and VIII of the cerebellum, as well as cerebral prefrontal, frontal, and temporal cortices [87]. On the other hand, the cerebro-ponto-cerebellar (CPC) afferent pathway connects the cerebral and cerebellar cortices through the contralateral middle cerebellar peduncle (*brachium pontis*) [88]. The CPC displays prominent streamlines between cerebellar crura I-II and cerebral prefrontal and temporal cortices. In notable contrast to the CTC, the temporal lobe is the main cerebral source of streamlines in the CPC [88]. Lastly, the basal ganglia constitute an important part of the CCS in primates, reciprocally communicating with the cerebellum both directly and through diverse cerebral areas [104]. They display modular organization into nuclei [115] and are ideally placed within the CCS to facilitate automatization of learned behaviors [100]. The basal ganglia play an important role in the complementary learning modes within the CCS [101, 104], have connections with both the cerebellar and cerebral cortex, and have access to salience signals and generate reward signals [100].

## Adaptations of Non-motor Modules in the Cerebello-Cerebral System Over Primate Evolution

In addition to the coordinated and conserved expansion of the CCS over vertebrate evolution, illustrated over primate evolution [85, 86], in human ancestors [116], as well as in parrots and cetaceans [83, 84], modular adaptations to subcomponents of both the cerebral and cerebellar cortex may have further developed this neuroanatomical basis for non-motor functions, sub-serving species-specific cognition. In primates, adaptations of the CCS have been most intensively studied. Anthropoid evolution is not primarily characterized by brain size, but by the reorganization of brain areas [58]. Since the dimensions of the primate CCS are tightly linked to brain size increases [40], and this system may be an exemplary neuroanatomical substrate for mosaic non-motor adaptations [100, 101] (see also previous sections), it is not surprising that diverse adaptations of this system have been reported [83, 84, 97, 98, 117–120]. As neuronal tissues are metabolically expensive and compete with other tissues [121, 122], such disproportional increases or decreases in relative size of structures within the system argue for the adaptive and reproductive roles they confer [123].

### Cerebral Evolution Is Related to Cerebello-Cerebral Connectivity

In the following section, we argue that considering cerebral evolution from the perspective of CCS connectivity may be more fruitful than considering cerebral evolution in isolation. Supporting this notion, both long- and short-range CCS connections between the frontal cortex and cerebellum are involved in mediating cognitive processing speed in humans [124]. Whether this finding extends to primates in general warrants investigation. In primates, a general allometric trend for the PFC to scale to the power of 1.2 versus the rest of the neocortex, suggests PFC expansion is a general primate adaptation [125]. Whether human PFC volume follows the primate allometric trend or is exceptionally large remains an open question. Although some studies show that it is [73, 126–129], and that volumetric increase is due to asymmetric changes [130], others note that its volume is not exceptional relative to the primate allometric trend [74, 131]. The larger PFC in primates with at the very extreme humans [7, 9, 48, 51] could be sufficient to support emergent cognitive abilities through sheer neuron number and increased connectivity [7, 48], increased modular organization [132–135], and specializations of functional areas [79, 136]. Alternatively, cerebello-prefrontal connectivity may play a significant role.

Evolutionary expansions of cerebral areas of primate-specific networks within the CCS may be most tightly linked to evolution of the lateral cerebellum [39, 86, 97, 119, 137]. Together, a combination of developmental [67] and functional [69] constraints may maintain the cerebello-cerebral scaling relationship over the course of evolution and development [67–69, 97]. Mirroring evolution [138, 139], extensive postnatal expansion in cerebral areas may allow more substantial influence of relevant early-life experiences [138]. This may occur in concert with the lateral cerebellum [97] that shows a comparable postnatal developmental trajectory [140, 141], and shows a connected pattern of evolutionary expansion [119]. Chemical perturbations of cerebellar lobules in developing mice led to more severe deficits in motor, cognitive, and social tasks than in adult individuals [142]. Whether this developmental role of the cerebellum in normal social and cognitive development relies on CCS connectivity warrants further investigation. Comparing humans with *Homo neanderthalensis* shows that most significant neurocranial shape changes between them are also recapitulated shortly after birth in humans [143]. Such findings extending to *Homo erectus* would provide additional evidence that recent evolution is mirrored by the latter stages of human brain development [67, 68].

Cerebral association areas appear to be expanded in humans compared to macaques [138] — and to a lesser extent to chimpanzees [75] — whereas sensorimotor areas do not [75, 132, 138]. Together, CCS connectivity might be able to better explain the relative expansions of the temporal [144–146] and parietal [138] cortices in humans, which are also supported by clear sulcal imprints on an early hominid endocast [147]. The basal ganglia, involved in reinforcement learning, habit learning, and action selection [101, 106, 109, 148] consist of separate motor, affective, and cognitive modules [104, 109]. These conserved subcortical nuclei [106, 115] also show volumetric differences between rats and primates [115]. Primate white matter architecture has likely also changed in a modular fashion, as the terminations of the arcuate fasciculus in humans cannot be predicted from cortical expansion and relocation versus macaques and chimps, whereas those of other major axon bundles can [78]. Extensive connectivity of this axon bundle with the temporal cortex in humans [149], but not in macaques and chimpanzees, may be an adaptation that serves human language function [150]. The relative shift to PFC bundles in the cerebral peduncle [107], an important CCS relay, is another such human-specific adaptation. The remapping factor, relating volume of association areas versus input areas [127], offers evidence for adaptation based on function: the primate neocortex is expanded relative to the dorsal thalamus, the primary sensory relay center of the CCS [136]. Evaluating evolutionary dynamics of the CCS in a connectivity-driven manner may help understand the functional relevance of adaptations within it [109].

Altogether, primate cerebral evolution in concert with the lateral cerebellum [97, 118, 119, 151] is characterized by the expansion of distributed association areas [86, 97, 132]. The CCS seems to provide a strong neuroanatomical scaffold, as CCS adaptations seem to be mirrored across primate evolution and human development [67–69, 97]. Both adaptations that are general among vertebrates [83–86, 97] and adaptations perhaps more specific to primates [138], *Hominoidea* [118], *Hominidae* [10], or humans [107, 119, 130], have been observed. Primate sub-lineage or species-specific adaptations noted in the cerebral cortex [75, 132, 138] are in many cases related to distinct CCS connectivity [81, 89, 97, 107, 141]. Future comparative analyses of areas delineated based on CCS connectivity may shed further light on the functional relevance and specifics of primate and human adaptations. The primate CCS may be compared to other mammals (e.g., cetaceans and elephants) and avian species (e.g., corvids and pigeons) prevailing in tasks with high cognitive demands, for example those requiring abstract reasoning [92–95]. Exchange of data and resources between scientists will be essential to move this endeavor forward [152, 153].

### The Cerebellum Is Relatively Large in Great Apes and Humans

Despite recent reports [7] suggesting that the cerebellum does not expand with total brain volume [154, 155], indications of cerebellar expansion over primate evolution have been long-present [85, 156]. These findings suggest that the cerebellum is at least equally worth considering as the cerebral cortex for the adaptive advantage it conveys over primate evolution. Great apes including humans have even broken from the primate evolutionary trend for the cerebral cortex and cerebellum to scale in tandem, having significantly larger cerebella [131]. Especially the posterolateral cerebellar hemispheres (i.e., the neocerebellum) have greatly increased in size in hominoids [118] and other vertebrates [97]. Although primate *general intelligence* (G) [157] is an evolutionarily labile trait, its evolutionary dynamics were best estimated by cerebellar volume relative to body size in a study of neuroanatomical measures [158]. A similarly disproportionately large cerebellum has been noted in dolphins [98, 120] and elephants [117].

Furthermore, in line with the extensive folding of the cerebellum into increasingly tight structures (from lobules to folia), the unfolded and flattened surface of the human cerebellum has close to 80% the surface area of the cerebral cortex [159].This contrasts with the surface area of the cerebellum in the macaque monkey, which is only 33% of that of the cerebral cortex, suggesting that cerebellar surface area expansion greatly exceeds that of the cerebrum [159]. Primate cerebellar foliation is associated with tool use [160]. In birds, cerebellar foliation correlates with nest complexity [161]. Both findings suggest that cerebellar surface area is related to cognitive ability. Additionally, endocast data indicate that compared to human ancestors, the great ape cerebellum has expanded relative to the neocortex [116]. These endocast data sidestep the common problem of brain size inference from endocasts by calculating the relative volume of the posterior cranial fossa — that houses the cerebellum — to whole endocast volume [116]. In sum, it appears that in humans the cerebellum has increased its size relative to that of the brain and the neocortex within the primate lineage, suggesting that adaptive advantages may be imposed by these changes. The relative growth of the cerebellum argues that apart from constraints — either neurodevelopmentally [67] or functionally [69] — acting on the system, also modular changes may occur within it [61, 82], as illustrated by divergent evolution of relative lateral cerebellar volumes across different groups of vertebrates [97].

### Distinct Cerebellar Adaptations Across the Vertebrate Lineage

Connectivity patterns within the primate CCS seem to predict concerted evolution of its constituents [85]. They may set the stage for the key evolutionary adaptations of the system over the primate lineage, and in doing so, may provide a functional and developmental neuroanatomic foundation for processes associated with internal mental capacities. Although evolution of the system seems to be partially constrained by this neuroanatomical foundation, mosaic changes [61] also occur. Comparative research has led to the present view that especially the expansion of the posterolateral cerebellar hemispheres and their reciprocal connections to the cerebral cortex may have been critical for the emergence of complex non-motor skills [16, 81, 118, 162]. The neocerebellar areas that have primarily expanded in anthropoids are crura I-II, as the relative areas of these structures increase in the evolution between macaques, chimpanzees, and humans [119]. A homologous area is already present in rats and mice, where it is called crus I [163]. This area is also referred to as the ansiform area across these species, and analysis of the volume fraction of this region versus the whole cerebellum again reveals an increase in great apes, and especially humans [151]. In human resting-state functional connectivity analysis, crura I-II, along with lobule VIIa, appear to belong to the cerebellar supramodal zone, through effective connectivity with the PFC, frontal pole, and inferior parietal lobe [164]. Prefrontal-projecting crura I-II are activated by purely symbolic visual representations of actions, which is ascribed to higher-order cognitive function associated with PFC [165]. In dolphins, who have a cerebellum that is even larger relative to whole brain volume than the human cerebellum, these lobules are not expanded as they are in humans [119, 120]. Even though dolphins are exposed to a vastly different external milieu, may have evolved independently from primates since shortly after the Cretaceous-Paleogene split 65 million years ago [166], and have brains that are organized much differently, they display similar cognitive abilities [92]. Dolphins lack a primate-like PFC and have a differently organized cerebellum [119, 120]. Cerebellar microzones have also undergone changes: in primates, wide D zones lead to expanded ansoparamedian areas, whereas cetaceans have large paraflocculi due to relatively large C2 zones [36, 120, 167, 168]. Differential prominence of cerebellar zones further supports the notion that divergent cerebellar lobular expansions may be related to connectivity of distinct cerebellar (micro)zones with function-related modules in the other components of the CCS. Further comparisons of the CCS between primates and cetaceans may prove useful for understanding its role in cognitive ability.

Direct comparison of the fiber bundles in the cerebral peduncle — a structure where cerebral-to-cerebellar fibers converge — revealed that the connections running from the PFC to crura I-II have become more prominent versus motor-bundles in humans relative to macaques [107]. It should be noted that the relative contribution of the posterior parietal cortex did not change [107]. For both the afferent CPC and efferent CTC pathways, tractography-based streamlines have been reported to be very prominent in crura I-II, as well as in the PFC and temporal cortices [87, 88]. Although no directly equivalent data exists in NHPs, these results support the general view that in humans, crura I-II have become strongly connected to the PFC and temporal cortex, with a general shift to transmodal associative connectivity between the cerebellum and cerebrum. A direct comparative observation that adds to the latter notion is that the ventromedial part of the DN — the main cognitive cerebellar output channel to the cerebrum — enlarges together with cerebral association areas [169]. In parrots, a similar CCS has developed and connectivity from cerebrum to cerebellum may have developed modularly versus other birds, through massive expansion of the medial spiriform nucleus, which acts as a chief relay between the forebrain and the cerebellum. Its volume is correlated with that of the telencephalon in birds [84]. Therefore, similar CCS circuitry may support cognition in species that have evolved independently. Together, these findings lend support to the original hypothesis put forward by Leiner, Leiner, and Dow, which holds that the cerebellum is directly involved in non-motor functions analogous to its role in motor functions [20, 82, 170, 171]. Additionally, they provide evidence that not only constraints [67, 69], but also modular or mosaic [61, 82] adaptations have shaped the CCS in primates and other vertebrates.

## Neuroimaging Further Reveals Cerebellar Non-motor Functions

In recent years, the relevance of cerebello-cerebral connections as a neural basis for non-motor processes has been further illustrated by neuroimaging methods such as resting state functional magnetic resonance imaging (rs-fMRI) studies in humans. Although still faced by conceptual and methodological challenges such as physiological artifacts and low signal-to-noise ratios [172], fMRI can help understand functional activations of the cerebellum in relation to the cerebral cortex.

The first large-scale rs-fMRI study investigating cerebellar functional organization demonstrated the somatotopy of two motor representations in the anterior and posterior lobes of the cerebellum [90], which were consistent with the well-documented sensorimotor representations in monkey and cat cerebella [173, 174]. This in turn argues for strong conservation of these representations across mammalian phyla. In addition, rs-fMRI functional cerebellar maps corroborated structural anatomic evidence by revealing that crura I-II had the highest functional connectivity with transmodal cortical association areas [90]. Furthermore, it was shown that the cerebellum is part of the brain’s default mode (DMN), salience, and executive control networks, which further underscores the proposed role of the cerebellum in mental functions [90, 91].

These rs-fMRI findings have been advanced by constructing an even more detailed functional topographical map of the cerebellum, providing further evidence for a reproducible macroscale functional organization in the human cerebellum that is variable among individuals [175]. These functional mapping approaches have provided another perspective on a domain-general function of the cerebellum on all cerebral output [21, 81, 176]. Extensive scanning of ten healthy individuals showed that cerebellar functional activation systematically lags that of the cerebrum, indicating that it responds to cerebral input [177]. This temporal lag is most pronounced for the frontoparietal network, which is spatially overrepresented within the cerebellum [91, 177]. These observations hint at adaptation of the human cerebellum to support abstract cognitive processing in this transmodal network — that would require more extensive time lag for predictive coding by cerebellar internal forward models. In some respect, such cerebellar areas could be considered *cerebellar association areas*, as they share extensive functional [175, 177] and structural [87, 88] connectivity, and evolutionary expansion [118, 119, 138, 163] with cerebral association areas, and potentially occupy a top position in the functional hierarchy [89–91, 175].

Additional insight comes from an alternative approach, which considers the brain not from a modular perspective, but rather as consisting of organizational gradients, for example separating uni- and transmodal areas [178, 179]. Guell and colleagues have applied such gradient methods to components of the CTC pathway [87], including the cerebellum [89], DN [180], and thalamus [181]. Their work has further supported the role for the cerebellum in non-motor functions by demonstrating cerebellar functional connectivity gradients that span from motor to default-mode regions and task-unfocused to task-focused processing [89]. Furthermore, connectivity gradients for both intracerebellar and cerebello-cerebral functional connectivity reproduced the hierarchically organized triple non-motor representation as revealed by winner-takes-all functional connectivity between the cerebellum and cerebrum [89, 90]. Both studies agree with comparative studies in implying an essential role for the crura I-II areas in supporting non-motor functions. Distinct functional organization across the DN [180] further support the theory that the CCS has divergent output channels for motor and non-motor functions [81, 113]. Lastly, a recent review on cerebello-cerebral functional connectivity underscores the involvement of the neocerebellum, and especially crura I-II, in cerebral functional networks, such as saliency, control, default, and language networks [16]. The caveat that functional activations measured through blood-oxygen-level-dependent (BOLD) signal reflect different neuronal processes in the cerebellum than in the cerebral cortex [172], should however be noted.

## The Cerebello-Cerebral System in Mental Disorders

Inspired by primate and vertebrate evolutionary and human functional imaging accounts, in combination with clinical observations that emphasize cerebellar function stretching beyond the motor domain, a cerebellar role in mental disease [182] including mood disorders [183] and schizophrenia [184, 185] has been increasingly investigated. The importance of the CCS in mental disorders has been, for instance, illustrated in a large study that compared schizophrenia patients with controls. Cerebellar gray matter volumes were most strongly reduced in areas connected to frontoparietal cortices. Additionally, cortical thickness of these cerebral areas correlated with the decrease in cerebellar volume [186]. Moreover, functional connectivity analysis in a gradient framework revealed that the sensorimotor-transmodal organizational axis of the cerebellum was compressed in individuals with schizophrenia, indicating decreased cerebellar functional differentiation [187]. Additionally, hypoconnectivity between the cerebellum and cerebrum was demonstrated, providing a substrate for altered functional performance in patients with schizophrenia [187].

As part of the brain’s association networks, including the central executive network (CEN) and DMN [16, 89, 188, 189], crura I-II again seem to be of elevated importance. For example, right crura Ia and Ib showed reduced functional connectivity with different areas in the CEN and DMN in unmedicated bipolar disorder [190]. Several other studies have shown that these regions of the CCS, that are expanded over primate evolution [118, 119, 163], are susceptible to alterations related to bipolar disorder [191–193]. Transdiagnostic accounts of mental disorder, investigating common alterations (e.g., genetic or neural), or the *p-factor* [194, 195], have underscored a general role of the cerebellum in mental health [196], although this could not be replicated in a later study [197]. Additionally, alterations in structural integrity of white matter fiber pathways of the pons, linking the PFC and neocerebellum, have been robustly associated to the *p-factor* [196, 197]. Altogether, these findings in clinical populations provide complementary evidence for the theory that the CCS supports cognitive adaptations in primates.

## Disentangling Drivers of Primate Ansiform Area Evolution

We have reviewed convergent evidence for the role of the cerebellum, and especially primate lateral cerebellar areas crura I-II, in non-motor function. That these areas have expanded over primate evolution in support of cognitive demands appears plausible. This is evidenced by coevolution of these areas [119] with cerebral association areas [97, 132, 138] implicated in cognition, as well as expansion of functional modules within CCS relays [107, 115, 169]. fMRI activations of crura I-II in cognitive tasks including language and social cognition [15, 198, 199], and resting-state connectivity analyses in complementary winner-takes-all network [90, 175, 177] and gradient-based [89] approaches imply involvement of the ansiform area in transmodal networks and cognition. Moreover, these areas also show alterations in mental disorders [190–193]. That primate cerebellar volumes in general relate to cognition is further supported by the relationship between cerebellar folding and tool use [160] and by the correlation between residual cerebellar volume and primate *general intelligence* [157, 158].

It is however difficult to disentangle drivers of the specific hypertrophy of crura I-II. Since it is challenging to compare socio-cognitive demands across primates, studies have used proxies such as diet, social group size, home range size, and tool use, and studied their relation to brain size [99, 160]. To find clear ecological correlates of cerebellar and ansiform area volumes, significant challenges need to be overcome, which include the difference in quality of comparative data collection, low statistical power, and unknown intraspecies variability. These challenges may be reduced by combining several comparative datasets and using phylogenetic methods that can incorporate intraspecies variation and thus uncertainty in the error term of phylogenetic analyses [99]. The correlation of tool use and cerebellar folding [160] shows that there is much to learn from naturally occurring behaviors. Through development of more ecologically valid proxies of naturally occurring non-motor or cognitive demands that are testable across large, quality-controlled datasets of primates including humans, relationships of these factors with additional cerebellar data such as ansiform area volumes [119, 151] relative to their phylogenetic positions [62, 63] can be examined. In this way, models can be generated that show which combination of proxies for cognitive abilities may drive cerebellar and, more specifically, ansiform area evolution. These drivers may, for instance, come from a combination of social or dietary demands, behavioral innovations, or technical demands stemming from for example tool use, bedding requirement, or brachiation challenges as per the Technical Intelligence hypothesis [131, 160, 200–202].

## Future Directions in Comparative Neuroscience of the Cerebello-Cerebral System

Comparative studies have already substantially contributed to our understanding of the cerebellum and CCS, but several questions remain. First, it is yet unclear what areas in the CCS have evolved hyper-allometrically, that is, have become exceptionally large in humans or great apes versus other primate species, or even vertebrates in general. This controversy is in substantial part due to lack of agreement over statistical methods used [203, 204]. Another reason is the lack of consistent delineation of the areas studied [203, 204]. Additionally, how the primate CCS may relate to that of other vertebrates with far-reaching cognitive abilities, remains underexplored. Comparative neurosciences would benefit from further improving statistical methods and community-wide guidelines, and systematic sharing of data and code which could alleviate analytical controversies in the future and expand the scope of statistical comparisons. Further, perhaps reconsideration of phylogenetic analysis tools is warranted [205]. Secondly, as this review illustrates, CCS connectivity may be an important anatomical [56, 70] and functional [69] scaffold that influences primate brain variations across evolution [40, 61]. More generally, we suggest that comparative analysis may be best performed between connected regions. Comparing primate structure scaling in a systematic, connectivity-driven manner may help establish the functional relevance of evolutionary variation and adaptation. For example, there are virtually no direct comparative studies on connectivity in the CCS [107]. Nonetheless, studying CCS connectivity is imperative for broadening our understanding of CCS function over evolution [87–90, 111, 175, 177]. Neuroimaging is a promising way to compare the phylogeny of the CCS and expansion of functional areas across primate species [206, 207], and beyond [83, 84]. Thirdly, it is important to combine data from multiple species with diverse proxies of brain structure, function, and behavioral variability [78, 208–210]. Defining a relevant common feature space would allow both cross-modal and cross-species comparison [210] and would greatly simplify comparative analysis of the CCS beyond measures of size alone. Finally, the complex anatomy of the cerebellum poses challenges. The cerebellar cortex is extremely folded and is difficult to image using MRI, including prominent physiological artifacts and low signal-to-noise ratio, and challenges in interpreting the meaning of BOLD signals [172]. Scanning at high field strengths and using dielectric pads may help to lessen the impact of these issues [211]. Moreover, integration of ultra-high-resolution post-mortem maps [212] with lower-resolution in vivo brain data may provide additional insights. More generally, we hope that with raised interest in cerebellar structure and function, the necessary novel conceptual and methodological approaches will be developed to account for cerebellar idiosyncrasies, rather than adopting approaches fit for the cerebral cortex to the cerebellum.

## Conclusion

Comparative studies across the vertebrate lineage provide a unique opportunity to increase our understanding of human brain evolution and function. With the current overview, we have provided an updated comparative evolutionary account that supports the view that the primate, and human cerebellum specifically, have undergone unique mosaic adaptations that have built upon the scaffold of the vertebrate CCS, based on developmental and evolutionary constraints [67, 69] and adaptive advantage [61, 70, 82]. Given the available evidence of substantial anatomical connectivity between the cerebellum and cerebral cortex in NHPs [111, 114] and humans [87, 88], and functional connectivity [89, 90, 175, 177], as well as functional activations in cognitive tasks or networks in humans [164, 198, 213], we argue that the CCS plays an important role in even abstract cognitive functions [13, 21, 39, 40, 61, 81, 82, 87, 88]. While the number of in vivo studies involving NHPs is still limited, the comparative approach offers a complementary research method to study cerebellar anatomy and functions in both NHPs and humans, especially when compared to other vertebrates with complex cognitive abilities such as Theory of Mind or mental attribution. Integrating multimodal, cross-species data in a standardized manner will enable the comparison of function-related, connectivity-dependent evolutionary variations across species [210]. Comparative neuroimaging methods are likely to contribute significantly to this endeavor [206, 207, 210], as illustrated by contemporary studies that compare macaques and chimpanzees to humans [6, 214–216]. Even without the ability to directly compare across species, the integration of evolutionary accounts with neuroimaging data [89, 175, 177] and insights into disease [197] allow for further exploration of cerebellar contributions to brain evolution and its associated function.
